# Covalent chemical probes

**DOI:** 10.1038/s42004-025-01652-6

**Published:** 2025-08-29

**Authors:** Keriann M. Backus, Andrew J. Wilson

**Affiliations:** 1 Department of Biological Chemistry, David Geffen School of Medicine, UCLA, Los Angeles, CA USA; 2 Department of Chemistry and Biochemistry, UCLA, Los Angeles, CA USA; 3 Molecular Biology Institute, UCLA, Los Angeles, CA USA; 4 DOE Institute for Genomics and Proteomics, UCLA, Los Angeles, CA USA; 5 Eli and Edythe Broad Center of Regenerative Medicine and Stem Cell Research, UCLA, Los Angeles, CA USA; 6 Jonsson Comprehensive Cancer Center, UCLA, Los Angeles, CA USA; 7 School of Chemistry, University of Birmingham, Edgbaston, Birmingham, UK

## Abstract

*Communications Chemistry* is pleased to introduce a Collection of research works focused on recent developments within the interdisciplinary field of Covalent chemical probes. Here, the Guest Editors highlight key themes and look towards the future of this research field.

What do aspirin, penicillin, omeprazole, and ibrutinib have in common? At first glance an anti-inflammatory drug, an antibiotic derived from Flemming’s penicillium mold, a proton pump inhibitor that blocks stomach acid production, and a kinase inhibitor to treat chronic lymphocytic leukaemia may simply seem like a disparate group of blockbuster drugs. However, this disparate group perfectly illustrates the power of covalency to effectively and safely treat wide-ranging illnesses. Thus, and often inspired by these breakthrough drugs, covalency has made a comeback both clinically and in basic research applications.

Chemical biomacromolecule labeling uniquely offers the ability to study and manipulate biology. Chemical probes with a covalent mode of action represent powerful tools that can be used for biology discovery, target validation (or off-target identification), and as starting points for drug discovery programmes. Bio-orthogonal chemistries alongside methods that harness the biosynthetic machinery to precisely integrate reporter functionality into biomacromolecules similarly facilitate our collective navigation of the dynamic cellular interactome to unveil new mechanistic understanding of biological processes, opportunities for intervention and new therapeutic modalities. In turn, this has stimulated development of new regioselective chemistries, computational methods and analytical methods to probe the complex environment of the cell. Collectively, this offers new opportunities to modulate, track and isolate proteins of interest in/from the complex cellular milieu. Covalent compounds are safe, efficacious, and useful for wide-ranging therapeutic applications, spanning cancers, autoimmune disorders, and infections. Covalency also uniquely enables functional biology, spanning discovery of new post translational modifications via proteomics, trapping of non-covalent interactions via latent electrophiles, including for both small molecules and biomolecules, and even the discovery and optimization of hyper-potent biologics that function *via* irreversible tethering of various therapeutic modalities to their targets.

Casting a broad net, in this Collection, we present a selection of manuscripts that capture the current state of research in covalent probes. This Collection begins with chemistries tailored to enhance covalency, including new electrophiles and their applications. Then, by showcasing the technical advances of combining proteomics with covalency, our set of chemoproteomic studies showcase the current state-of-the art for target deconvolution and mode-of-action studies, enhanced by new reagents and platforms. Lastly, we turn to the biological applications of covalency, spanning protein, aptamers, and glycan interactomes. Taken together these studies unveil new mechanistic understanding of biological processes, opportunities for intervention and new therapeutic modalities and showcase the unique strengths of covalency to enable high throughput biochemistry, chemical probes and drug discovery.

## Covalent chemistry for ligand and drug discovery

Covalent chemical probes are bioactive ligands that form a covalent bond with their target biomacromolecule(s). Drugs with a covalent mode of action have been known and used for over a century, although historically, pursuit of such bioactive compounds has been avoided due to concerns over lack of selectivity. Over the last three decades however, the use of ligands bearing reactive handles has seen increased interest spawning efforts to rationally design covalent drugs and new approaches to study protein function such as activity-based protein-profiling. Where covalent bioactive ligands are concerned, increased selectivity and duration of action represent advantages. In this Collection, exciting developments are described for small-molecule growth factor inhibitors^1,2^, immunomodulatory glycolipids^3^, E3 ligases^4^, and peptide-based inhibitors of PPIs^5^& viral targets^6^. Powerful new methods based on sulfur-fluoride exchange are facilitating inhibitor discovery for challenging molecular and disease targets e.g., phosphodiesterases^7^ and *T. Brucei*^8^. Alongside this progress, new methods are facilitating rapid discovery of inhibitors by integrating labeling chemistries with biological selection^9,10^, high-throughput plate-based synthesis and screening^11^, and mapping covalent chemistries to ever more diverse ligand types such as aptamers^12^. Underpinning these efforts are the development of new chemistries that may act as amino acid side chain warheads^13,14^. Finally, computational methods development is accelerating development of covalent inhibitors^15^.

## Chemical proteomics

Chemical proteomics involves the use of chemical probes to study the proteome. It can be enormously powerful in target and off-target identification. This is exemplified with reagents that profile palmitoylation^16^, ligand identification for monoacylglycerol lipids^17^ and photoaffinity profiling of pharmacophores for kinase inhibitors^18^. The approach can be similarly powerful for profiling binding sites^19^. Finally, the ability to effect controlled temporal or organelle specific activation of chemical probes offers opportunities to resolve signaling pathways with much greater precision^20^. Underpinning these efforts are studies to understand the fundamental reactivity of covalent labeling chemistries in the cell e.g., thiol-ene chemistry^21^ and new reagents to facilitate higher resolution analyses of proteomes^22^.

## New tools and covalent chemistry for biology

The opportunity to be creative with new synthetic methods and workflows for biology draws synthetic chemists to the arena of chemical biology. Bio-orthogonal chemistry received the Nobel prize in 2022, however there remains a need for improved and new biorthogonal reactions^23–25^, to facilitate new approaches for proteomics, imaging and proximity-induced workflows like “traceless” protein labeling^26,27^ and controlled protein assembly^28^. Even small modifications incorporated through metabolic labeling e.g., isotopes offer promise to delineate signaling specificities^29^, whilst click chemistry can be used to induce labeling of low affinity glycan ligands through chelate co-operativity^30^, and detect biological processes such as NETosis through turn-on fluorescence^31^. Underpinning these efforts are the development of multifunctional reagents derived from unsaturated saccharides which can react with cysteine and release carboxylic acids^32^ and methods for assembly of proteins bearing site specific complex post-translational modifications^33^.

## Outlook

To conclude, we hope to also inspire ongoing and future efforts to further enhance covalent chemistries. As aspirin and penicillin revealed, covalent molecules and chemistries are ubiquitous, with many still likely waiting to be discovered, hidden in screening decks, natural products, and metabolites, around the globe. Enabled by a high-powered emerging suite of technologies, pinpointing which molecules could be covalent and what proteins (or other biomolecules) they label has never been easier. That being said, covalents do pose unique challenges that remain to be fully explored. In some cases, ultra-long half-lives of covalents, intimately tied to the protein of interest, can raise concerns about idiosyncratic toxicity, as does the possibility of scavenging endogenous redox active cofactors, such as glutathione. We urge the field to take care when progressing new covalent chemotypes and to rigorously characterize both the specific and more generalized physiologic effects of each screening hit. This rigor, together with the ever-evolving new technologies, chemistries, and creative applications, will ensure a bright future for covalent probes across drug, chemical probe, and molecular mechanistic studies. We hope researchers will continue to be inspired by all aspects of covalency, spanning fortuitous discoveries and the unexpected covalent mechanisms through to next generation clinical candidates and breakthrough drugs.

## References

[1] Morese, P. A. et al. Factors affecting irreversible inhibition of EGFR and influence of chirality on covalent binding. *Commun. Chem.* **8**, 111 (2025). 40204983 10.1038/s42004-025-01501-6 PMC11982232

[2] Chen, X. et al. Structure-based design of a dual-warhead covalent inhibitor of FGFR4. *Commun. Chem.* **5**, 36 (2022). 36697897 10.1038/s42004-022-00657-9 PMC9814781

[3] Lee, J., Son, S., Lee, M. & Park, S. B. Development of potential immunomodulatory ligands targeting natural killer T cells inspired by gut symbiont-derived glycolipids. *Commun. Chem.* **8**, 98 (2025). 40169880 10.1038/s42004-025-01497-z PMC11961698

[4] Maspero, E. et al. Structure-based design of potent and selective inhibitors of the HECT ligase NEDD4. *Commun. Chem.* **8**, 164 (2025). 40437006 10.1038/s42004-025-01557-4 PMC12119865

[5] Cai, G. et al. Design of a covalent protein-protein interaction inhibitor of SRPKs to suppress angiogenesis and invasion of cancer cells. *Commun. Chem.* **7**, 144 (2024). 38937565 10.1038/s42004-024-01230-2 PMC11211491

[6] Medrano, F. J. et al. Peptidyl nitroalkene inhibitors of main protease rationalized by computational and crystallographic investigations as antivirals against SARS-CoV-2. *Commun. Chem.* **7**, 15 (2024). 38238420 10.1038/s42004-024-01104-7 PMC10796436

[7] Zhao, X. Z. et al. Targeted sulfur(VI) fluoride exchange-mediated covalent modification of a tyrosine residue in the catalytic pocket of tyrosyl-DNA phosphodiesterase 1. *Commun. Chem.* **7**, 208 (2024). 39284936 10.1038/s42004-024-01298-w PMC11405833

[8] Mantilla, B. S. et al. Discovery of Trypanosoma brucei inhibitors enabled by a unified synthesis of diverse sulfonyl fluorides. *Commun. Chem.* **7**, 237 (2024). 39427042 10.1038/s42004-024-01327-8 PMC11490619

[9] Mathiesen, I. R., Calder, E. D. D., Kunzelmann, S. & Walport, L. J. Discovering covalent cyclic peptide inhibitors of peptidyl arginine deiminase 4 (PADI4) using mRNA-display with a genetically encoded electrophilic warhead. *Commun. Chem.* **7**, 304 (2024). 39702664 10.1038/s42004-024-01388-9 PMC11659602

[10] Wu, Y. et al. Identification of photocrosslinking peptide ligands by mRNA display. *Commun. Chem.* **6**, 103 (2023). 37258712 10.1038/s42004-023-00898-2 PMC10232439

[11] Vuorinen, A. et al. Enantioselective OTUD7B fragment discovery through chemoproteomics screening and high-throughput optimisation. *Commun. Chem.* **8**, 12 (2025). 39809917 10.1038/s42004-025-01410-8 PMC11732987

[12] Soxpollard, N., Strauss, S., Jungmann, R. & MacPherson, I. S. Selection of antibody-binding covalent aptamers. *Commun. Chem.* **7**, 174 (2024). 39117896 10.1038/s42004-024-01255-7 PMC11310417

[13] Zhao, Y. et al. Catalyst-free late-stage functionalization to assemble α-acyloxyenamide electrophiles for selectively profiling conserved lysine residues. *Commun. Chem.* **7**, 31 (2024). 38355988 10.1038/s42004-024-01107-4 PMC10866925

[14] Columbus, I. et al. Species-specific lipophilicities of fluorinated diketones in complex equilibria systems and their potential as multifaceted reversible covalent warheads. *Commun. Chem.* **6**, 197 (2023). 37715018 10.1038/s42004-023-01004-2 PMC10504258

[15] Holcomb, M., Llanosa, M., Hansel-Harrisa, A. & Forlia, S. Structure-based rational design of covalent probes. *Commun. Chem*. **8**, 242 (2025). 10.1038/s42004-025-01606-y PMC12343802 40797071

[16] Hsiao, W.-C. et al. Marine diterpenoid targets STING palmitoylation in mammalian cells. *Commun. Chem.* **6**, 153 (2023). 37463995 10.1038/s42004-023-00956-9 PMC10354091

[17] Shanbhag, K. et al. Chemoproteomics identifies protein ligands for monoacylglycerol lipids. *Commun. Chem.* **8**, 197 (2025). 40615626 10.1038/s42004-025-01589-w PMC12227648

[18] Korovesis, D., Mérillat, C., Derua, R. & Verhelst, S. H. L. Proteome selectivity profiling of photoaffinity probes derived from imidazopyrazine-kinase inhibitors. *Commun. Chem.* **8**, 34 (2025). 39910186 10.1038/s42004-025-01436-y PMC11799219

[19] Ábrányi-Balogh, P. et al. Mapping protein binding sites by photoreactive fragment pharmacophores. *Commun. Chem.* **7**, 168 (2024). 39085342 10.1038/s42004-024-01252-w PMC11292009

[20] Huang, K.-T. & Aye, Y. Toward decoding spatiotemporal signaling activities of reactive immunometabolites with precision immuno-chemical biology tools. *Commun. Chem.* **7**, 195 (2024). 39223329 10.1038/s42004-024-01282-4 PMC11369232

[21] Campaniҫo, A. et al. Chemical- and photo-activation of protein-protein thiol-ene coupling for protein profiling. *Commun. Chem.* **8**, 25 (2025). 39880982 10.1038/s42004-025-01412-6 PMC11779957

[22] Burton, N. R. & Backus, K. M. Functionalizing tandem mass tags for streamlining click-based quantitative chemoproteomics. *Commun. Chem.* **7**, 80 (2024). 38600184 10.1038/s42004-024-01162-x PMC11006884

[23] Gao, J. et al. Direct ring-strain loading for visible-light accelerated bioorthogonal ligation via diarylsydnone-dibenzo[b,f ][1,4,5]thiadiazepine photo-click reactions. *Commun. Chem.* **3**, 29 (2020). 36703431 10.1038/s42004-020-0273-6 PMC9814081

[24] Manicardi, A., Cadoni, E. & Madder, A. Hydrolysis of 5-methylfuran-2-yl to 2,5-dioxopentanyl allows for stable bio-orthogonal proximity-induced ligation. *Commun. Chem.* **4**, 146 (2021). 36697666 10.1038/s42004-021-00584-1 PMC9814669

[25] Versteegen, R. M. et al. Ortho-functionalized pyridinyl-tetrazines break the inverse correlation between click reactivity and cleavage yields in click-to-release chemistry. *Commun. Chem.* **7**, 302 (2024). 39702778 10.1038/s42004-024-01392-z PMC11659415

[26] Beard, H. A. et al. Photocatalytic proximity labelling of MCL-1 by a BH3 ligand. *Commun. Chem.* **2**, 133 (2019). 33763603 10.1038/s42004-019-0235-z PMC7610391

[27] Gómez-Santacana, X. et al. A modular click ligand-directed approach to label endogenous dopamine D1 receptors in live cells. *Commun. Chem.* **8**, 113 (2025). 40216891 10.1038/s42004-025-01504-3 PMC11992035

[28] Worthy, H. L. et al. Positive functional synergy of structurally integrated artificial protein dimers assembled by Click chemistry. *Commun. Chem.* **2**, 83 (2019).

[29] Trainor, N. et al. Tracking DOT1L methyltransferase activity by stable isotope labelling using a selective synthetic co-factor. *Commun. Chem.* **7**, 145 (2024). 38937590 10.1038/s42004-024-01227-x PMC11211345

[30] Nomura, S. et al. Cancer discrimination by on-cell N-glycan ligation. *Commun. Chem.* **3**, 26 (2020). 36703447 10.1038/s42004-020-0270-9 PMC9814842

[31] Ramos Cáceres, E., Kemperman, L. & Bonger, K. M. Environment-sensitive turn-on fluorescent probe enables live cell imaging of myeloperoxidase activity during NETosis. *Commun. Chem.* **7**, 262 (2024). 39533026 10.1038/s42004-024-01338-5 PMC11557929

[32] Dong, S. et al. Development of ketalized unsaturated saccharides as multifunctional cysteine-targeting covalent warheads. *Commun. Chem.* **7**, 201 (2024). 39251816 10.1038/s42004-024-01279-z PMC11385544

[33] Burlina, F. et al. Auxiliary-assisted chemical ubiquitylation of NEMO and linear extension by HOIP. *Commun. Chem.* **2**, 111 (2019). 31942456 10.1038/s42004-019-0211-7 PMC6962051

